# Human Fatalities Caused by Hornet, Wasp and Bee Stings in Spain: Epidemiology at State and Sub-State Level from 1999 to 2018

**DOI:** 10.3390/biology10020073

**Published:** 2021-01-20

**Authors:** Xesús Feás

**Affiliations:** Academy of Veterinary Sciences of Galicia, Edificio EGAP, Rúa Madrid, No. 2-4, 15707 Santiago de Compostela (A Coruña), Spain; xesusfeas@gmail.com

**Keywords:** epidemiology, X23, fatalities, venomous animals, Spain, stings, hornets, wasps, bees, *Vespa velutina*

## Abstract

**Simple Summary:**

Although not frequent, hornet, wasp, and bee stings may be life-threatening. Over the 20-year period studied, a total of 78 fatalities were recorded in Spain, the annual mortality rates ranging from 0.02 to 0.19 per million inhabitants. The fatal events mainly affected men over 65 years of age, and took place in summertime, at “unspecified places”. At regional level, the deaths tended to concentrate in three communities: Galicia, Andalucía, and Castilla y León. Surprisingly, Galicia showed high mortality rates in hornet stings. The implication of the invasive species *Vespa velutina,* also known as the Asian hornet, is examined. In light of the findings, there is evidence to consider the health-related importance and consequences of *Vespa velutina*.

**Abstract:**

Epidemiology of fatalities in Spain due to hornet, wasp, and bee stings (Cause Code of Death: X23) is described. Over a 20-year period (1999–2018), a total of 78 fatalities were recorded, mostly occurring in males (85.9%), of 65 years and older (52.6%), at “unspecified places” (67.9%), and in the months of July and August (50%). The X23 mortality rates (X23_MR_) expressed in terms of annual rates and per million inhabitants, varied from 0.02 to 0.19 (mean value ± standard deviation = 0.09 ± 0.05), placing Spain at low levels in comparison with other countries. A more detailed and specific breakdown of the distribution of the yearly deaths at the sub-state level and across communities reveals some striking features. They were more concentrated in the communities of Galicia (35.8%), Andalucía (21.7%), and Castilla y León (12.8%). X23_MR_ were estimated in Galicia at 1.82, 1.10, and 2.22 in 2014, 2016, and 2018, respectively; and in Asturias at 1.88 and 0.97, in 2014 and 2017, respectively. The role of the invasive species *Vespa velutina* (VV) is examined. Due to its habits, abundance, and broader distribution, the risk that VV represents to human health is unmatched by other Hymenoptera native species.

## 1. Introduction

Insects are important and dominant creatures in nature because of their diversity, ecological role, and influence on natural resources. In fact, insects originate the biological foundation for all terrestrial ecosystems, and every living creature relies, in one way or another, on insects for equilibrium [1]. Certainly the role of insects in human evolutionary history and well-being is indisputable [2].

Social bees and wasps are recognized as important for early hominids. The practice of harvesting wild bees for honey and wax, as well as hunting wasp colonies for brood and nests, survives in our day, although man has now become a beekeeper and an incipient wasp breeder. Beehive harvested products (honey, pollen, propolis, bee bread, wax, royal jelly, and venom) have been useful since ancient times, not only as food but also as treatment for medical conditions [3,4,5,6,7,8,9,10,11,12,13]. Wasps too are appreciated as nutritious food and their nests are used therapeutically in traditional medicine [14]. Bees and wasps provide an important service to ecosystems, contributing to the improvement of biodiversity while helping to maintain ecological balance.

Moreover, their medical-veterinary importance should also be outlined. Among invertebrate groups, the Hymenoptera represent the only species possessing inoculator’s venom, produced by the venom gland. In the Aculeates, the defining feature is that the egg-laying ovipositor is modified to form a sting [15]. The major insect group causing reaction are those of the genera *Apis* (honeybees) and *Bombus* (bumblebees) in the family Apidae; *Vespula* (yellowjackets), *Dolichovespula* and *Vespa* (hornets), and *Polistes* (paper wasps) of the family Vespidae; and different stinging ants of the superfamily Formicoidea, such as Solenopsis (fire ants) and Pogonomyrmex (harvester ants) [16].

A&E physicians encounter large numbers of hymenopteran sting cases each year. In fact, during their lifetime, 94.5% of people are stung by wasps, honeybees, hornets, or bumble-bees, resulting in an unpleasant experience [17]. Insects are not prone to attack and sting humans; however, social Hymenoptera such as hornets, wasps, and bees: (i) have a massive defensive response to any threat to the colony and (ii) the evolution of the venom system in social Apidae and Vespidae appears to have further evolved to cause pain and augment the immune response in humans and other vertebrate predators [18].

There are five types of reactions to Hymenoptera stings including normal local reactions (NLR), large local reactions (LLR), systemic anaphylactic reactions (SAR), systemic toxic reactions (STR), and unusual reactions (UR) [19]. Most stings by Hymenoptera species only lead to a NLR: redness, swelling, itching, and pain. Yet, in a minority of cases (0.02% to 4%) a SAR, sometimes life-threatening, may occur [20]. The prevalence of SAR in epidemiological studies in Europe to hymenoptera stings ranged between 0.3 and 7.5% in adults and 0.15–3.4% in children [21]. Despite the common event of hymenopteran stings, about 100 million cases are detected each year around the world, fatalities due their stings are statistically rare [17]. Under the current International Classification of Diseases and Related Health Problems 10th Revision (ICD-11), Chapter XX allows the classification of environmental events and circumstances as the cause of injury, poisoning, and other adverse effects. Fatalities due to hornet, wasp, and bee stings (including yellow jackets) are coded as single category, the X23 [22].

The reviewed incidence of insect-sting mortality around the world is statistically low, but not negligible, ranging from 0.03 to 0.48 fatalities per million inhabitants per year [23]. In Spain, the data is scarce. The Spanish Society for Allergology and Clinical Immunology indicates the mortality rate at 0.08 per million inhabitants per year, which means that around 3 or 4 people could die each year from this cause in the event that after sting they did not receive immediate medical attention [24]. Similar values are cited by other authors, with 3 deaths per year (0.08 per million inhabitants and year between 1983 and 1993) [25]. It was highlighted that fatalities due to stinging insects are poorly registered and documented. Furthermore, post-mortem studies suggest that fatal hymenoptera allergy may be underestimated [26,27].

Due to its complex orography and geographic situation, there are marked biogeographical differences in terms of species, populations, and ecosystems throughout Spain. Spain is the most climatically diverse country in Europe, with 13 different Köppen climates. With four biogeographical regions (Mediterranean, Atlantic, Alpine, Macaronesian) several habitats are present to which a variety of hymenopteran communities is associated. In the Iberian Peninsula, just over 9000 species have been cited, which represents 6.13% of the world’s fauna. In fact, Spain possesses one of the richest faunas of hymenopterans in the world [28]. An account of bee species and subspecies recorded in Spain list around 1105 [29]. Current statistics regarding the numbers of apiarists active in Spain show in 2018 a total of 2,868,337 hives registered, which means an average of 5.7 beehives/km^2^ and 6.1 beehives per 100 inhabitants. The Vespidae family has four subfamilies on the Iberian Peninsula and a total of 162 species are registered so far: 133 Eumeninae, 11 Masarinae, seven Polistinae, and 11 Vespinae [30].

In the last ten years, three not-native species in the genus Vespa Linnaeus, 1758 have been detected in Spain: (i) the yellow-legged Asian hornet (*Vespa velutina* Lepeletier 1836), found in 2010 in the community of Navarra [31]; (ii) the black shield hornet (*Vespa bicolor* Fabricius, 1787) found in 2013 in Málaga (community of Andalucía) [32] and the Oriental hornet (*Vespa orientalis* Linnaeus 1771) found in Valencia (community of Valencia) in 2012 [33]. The three species have been able to fund stable populations in the entrance areas; however, they differ in their propagation capacity, highlighting the invasive nature alien species (IAS), the *Vespa velutina*.

Accidents due to insect stings, mainly hornet, have been reported in local and national newspapers and other media such as TV, suggesting that the number of people affected might be increasing. In this context of certain social alarm, in 2015, it was communicated that from a strictly medical point of view, there is no reason for the social alarm generated by the appearance of the Asian hornet in the Spanish hymenoptera population [24]. In 2016, Galician sanitary authorities declared that the “Asian hornet is highly important regarding both economy and biodiversity, not so much regarding public or clinical health” [34]. The Asian hornet was first detected in Europe, in 2004, concretely in France, where studies from French poison control centers show that the increase in the Asian hornet population in the south west region of the country has not thus far resulted in an increase in the number of Hymenoptera stings between 2004 to 2008 [35].

Health risks and deaths caused by wasp stings have become a public health concern around the world. In China, data from Hubei province show that fifty-four people died, according to the clinical data of hospitalized wasp sting patients (*n* = 1091) from 2009–2011 [36]. In South Korea from 2010 to 2014, there were 483,233 calls requesting removal of wasp nests and Hymenoptera stings caused 78,860 injuries and 49 deaths, *Polistes rothneyi koreanus* Vecht and *Vespa velutina* being the most prevalent sources [37]. A recent global appraisal on the epidemiology of *Vespa* spp. sting highlights that, despite the number of cases present, this health problem could be underestimated due to the lack of public information [38].

There is a general lack of data regarding the fatalities caused by insect stings in Spain. Therefore, the aim of the present work is to document and characterize the Spanish deaths due to hornet, wasp, and bee stings over a 20-year period (1998–2018) at the state and sub-state level. The implication of the invasive species *Vespa velutina* is examined. In the light of the findings, I argue that in Spain there are spots of extremely high exposure to insect stings, mainly due to the *Vespa velutina*. It is necessary that health issues are seen as a core part of the impact associated with this invasive alien species.

## 2. Materials and Methods

The Spanish National Statistical Institute (INE) was queried about all fatalities in Spain resulting from contact with hornets, wasps, and bees (ICD-10 code X23). The INE is a legally independent administrative autonomous institution assigned to the Ministry of Economy, Industry, and Competivity via the Secretary of State for the Economy and Business Support. The INE is responsible for assembling causes of death as well as other epidemiologic data from death certificates in Spain.

The INE returned a microdata file, comprising individual data for all X23-related fatalities registered between 1999 and 2018, both included. Observations were filtered appropriately to elicit anonymous information so as to ensure confidentiality. For each observation, the following particular variables were obtained and grouped:

– Gender.
– Month of occurrence.
– Place of occurrence, comprising nine categories: home; residential institution; school, other institutions, and public administrative areas; sports and athletics; street and highway; trade and services; industry and construction; farming; other specified places; and unspecified places.
– Age categories, comprising four ranges: children (0–14 years), youth (15–24 years), adults (25–64), and seniors (65 years and older).
– Community in which the fatality happened, comprising nineteen administrative territories: Andalucía, Aragón, Asturias, Balearic Islands, Canary Islands, Cantabria, Castilla y León, Castilla-La Mancha, Cataluña, Valencia, Extremadura, Galicia, Madrid, Murcia, Navarra, País Vasco, La Rioja, City of Ceuta, and City of Melilla.

The X23 mortality rates (X23_MR_) were calculated by dividing the number of people who died from a X23 cause (n_i_) by the number of people residing in the sample area as reported on the census (P_j_), as of 1 January of each year:

(1) $${X23}_{\mathrm{MR}}=\sum_{i\in s}n_{i}/P_{j}.$$

The X23_MR_ are expressed in terms of annual rates (i.e., per year) and per 1,000,000 inhabitants. The X23_MR_ were obtained both for the state at sub-state level, i.e., for the Spanish State as well as for each of the nineteen administrative territories. State and sub-state population rates were obtained from INE.

## 3. Results

**Figure 1:**
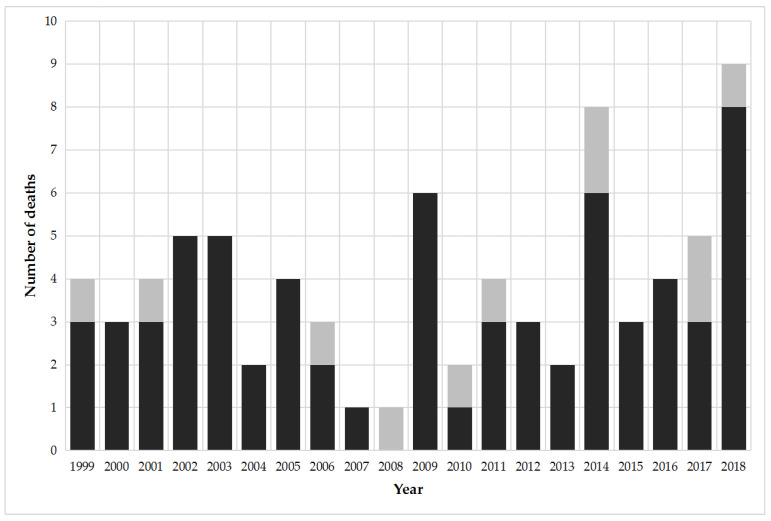
Annual number of deaths due to hornet, wasp, and bee stings in Spain (1999–2018) according to gender of victims (male in black and female in grey).

During the years 1999–2018, 78 deaths were officially registered in Spain with the external cause of injury code X23, i.e., deaths related to contact with hornets, wasps, and bees (Figure 1). It means an average number of 3.9 deaths per year during the 20-year period studied, reaching a high of nine deaths in 2018, and minimum values of one death in 2007 and 2008. Of the total number of victims, sixty seven were among men and eleven were among women. The fatalities accounted disproportionately for males with 85.9% of the total deaths.

**Figure 2:**
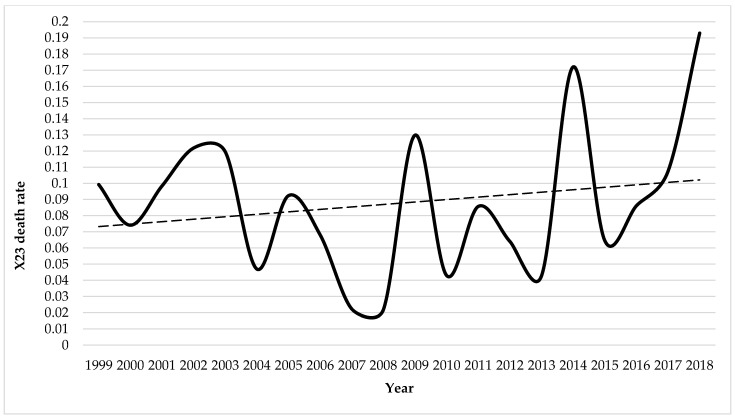
Annual mortality rates per million inhabitants due to hornet, wasp, and bee stings in Spain, 1999–2018.

The X23_MR_ values for the 20-year period analyzed ranged from 0.02 to 0.19 (mean value ± standard deviation = 0.09 ± 0.05) (Figure 2).

In relation to place of occurrence, fatal stings occurred in a wide range of situations, including: 7.6% at home; 1.2% at school, other institutions and public administrative areas; 5.1% on streets and highways; 8.9% at farms; and 8.9% at other specified places. However, the 67.9% of the fatalities are recorded at “unspecified places”. The most common age group to be fatally injured by hornet, wasp, and bee stings was 65 years and older, with a total of forty-one people, representing 52.6% of the deaths (Table 1). Next, the 25–64-year-old age group with thirty-seven deaths, representing 47.4% of the total number of deaths. No deaths were recorded among the other two age categories, children (00–14 years) and youth (15–24 years).

**Figure 3:**
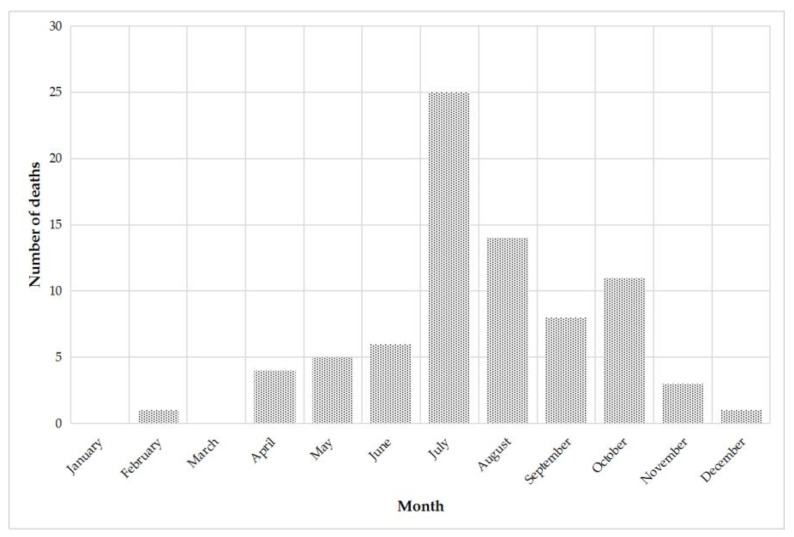
Fatalities due to hornet, wasp, and bee stings by month in Spain, 1999–2018.

Fatalities according to time of year rank, July as having the highest percentage of deaths caused by hornets, wasps, and bees, accounting for 32.0% of the total (Figure 3). Followed by August, October, and September, with a 17.9, 14.1, and 10.2% of the total deaths, respectively. No deaths occurred from 1999 to 2018 in Spain in the months of January and March.

**Figure 4:**
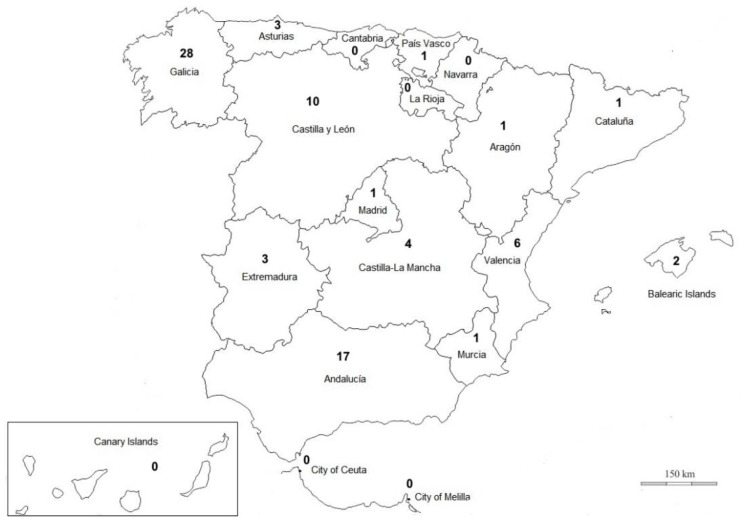
Distribution of fatalities caused by hornet, wasp, and bee stings in Spain at the sub-state level, 1999–2018.

A map containing the number of deaths at the sub-state level due to hornet, wasps, and bee stings based on the twenty-year period studied (1999–2018) is shown in Figure 4. There are no registered deaths due to hornet, wasp, and bee stings in the communities of Navarra, La Rioja, Cantabria, Canary Islands, City of Ceuta, and City of Melilla. Furthermore, Aragón, Cataluña, Madrid, Murcia, and País Vasco only registered one death over the period studied. The deaths were more highly concentrated in the communities of Galicia (35.8%), Andalucía (21.7%), and Castilla y León (12.8%), with twenty-eight, seventeen, and ten victims, respectively.

X23 mortality rates (X23_MR_) at the sub-state level varied across the territory (Table 2). High X23_MR_ values were found for Asturias (1.88 in year 2014), and for Galicia (1.82 in 2014, 1.10 in 2016, and 2.22 in 2018)

## 4. Discussion

The majority of deaths (*n* = 78) in Spain over the 20-year period (1999–2018) due to hornet, wasp, and bee stings can be described as occurring in males (85.9%), in the age group of 65 years and older (52.6%), at “unspecified places” (67.9%), and in the months of July and August (50%).

The role of gender in the pathogenic mechanisms of anaphylaxis in general, and of insect-venom anaphylaxis in particular, is still largely unknown. It was suggested that the higher incidence of anaphylaxis to venom in men can probably result from differences both in occupation and degree of exposure. Occupational anaphylaxis has been reported especially for beekeepers, beekeeping being an activity mainly carried out by men. Moreover, since more men than women work outdoors and do sports, they are stung more frequently than women and might therefore be at a higher risk of allergic reactions. Mastocytosis, a risk factor of venom allergy, prevails in males rather than females [39]. Despite theoretical premises and certain clinical observations indicating estrogen as having an important role in allergic diseases, the influence on hypersensitivity to stinging insects’ venom is not unequivocal and remains still open. Other investigators, conversely, found out that women are more often affected by Hymenoptera Venom Allergy (HVA) than men [40]. Gender is one of the risk factors in the reaction to fatal stings, being men three times more at risk than women [41]. Regarding age, there are differences in hymenopteran-induced fatalities. The number of deaths clearly evidenced the higher risk in the elderly. Comparable observations were described in Colombia, where the 50-year-and-older group accounted for the 73% of the fatalities [42] and in South Korea, where deaths occurred in those over the age of 50 [37]. However, it was highlighted that a clinically important number of children do not outgrow allergic reactions to insect stings, and there is a prolonged benefit in children to the use of venom immunotheraphy, leading to lower risks of systemic reactions to stings [43]. Fatalities due to hornet, wasp, and bee stings in Spain were concentrated in summer (July and August) as well as late spring and early fall. In fact, stinging insect colonies are typically at their maximal size in late summer and/or autumn, with ranges varying depending on the species. Bumblebees, wasps, and hornets are eusocial insects with a colony-based annual life cycle and a caste-based social system, with queens, workers, and males reaching peak abundance at different stages in the cycle.

In these species, there is no colonial life in winter. Old queens, workers, and males die in the autumn or winter, and only the newly mated queens survive. New queens hibernate and start new nests and colonies next spring. In winter, beehives show few or no signs of activity. Inside, there are a few thousand workers forming a cluster with the queen bee. As a result of this increased activity both in insects and humans doing outdoor activities, the possibility of coming into contact with a stinging insect becomes much greater during the late summer. Related to the place of occurrence, fatal stings occurred in a wide range of situations, including: 7.6% at home; 1.2% at school, other institution, and public administrative areas; 5.1% at street and highway; 8.9% at farm; and 8.9% at other specified places. However, the 67.9% of the fatalities are recorded at “unspecified places”. The frequent use of the above imprecise category gives little insight into the circumstances and the contexts in which such events occur, limiting the development of prevention initiatives.

At state level, the X23_MR_ values of the 20-year period analyzed ranged from 0.02 to 0.19 (mean value ± standard deviation = 0.09 ± 0.05), placing Spain at lower levels, compared to the annual mean data of deaths per million inhabitants in other countries (Table 3). Values equal to or less than 0.08 for the whole of Spain are recorded in half the years corresponding to the study period, specifically 0.02 in 2007 and 2008, 0.04 in 2010 and 2013, 0.05 in 2004, 0.06 in 2012 and 2015, 0.07 in 2000 and 2006, and 0.08 in 2011.

A more detailed and specific breakdown of the distribution of the deaths due to stinging insects at the sub-state level by year and across communities reveals some striking features. The communities concentrating the high beehive stock in Spain show a decline of X23_MR_ in the last ten years of the period studied (2009–2018). A very high rate of insect sting fatalities involving residents of Galicia and Asturias occurred in recent years (2014, 2016, 2017, and 2018).

Compared to the first decade of the study period analyzed, the second (2009–2018) saw a reduction in the number of sting-related fatal cases in: Andalucía, Aragón, Castilla y León, Cataluña, Valencia, Extremadura, and Madrid; and the opposite trend with an increase in X23_MR_ in: Asturias, Balearic Islands, Castilla-La Mancha, Galicia, Murcia, and Pais Vasco. Out of the total Spanish beehive stock, 21.8% (15.4 hives/km^2^; 58.3 hives/100 inhabitants), 19.6% (6.4 hives/km^2^; 6.7 hives/100 inhabitants), 15.3% (4.7 hives/km^2^; 18.2 hives/100 inhabitants), and 12.5% (15.4 hives/km^2^; 7.2 hives/100 inhabitants) are distributed in Extremadura, Andalusia, Castilla y León, and Valencia, respectively [52].

A very high rate of insect sting fatalities involved residents of Galicia and Asturias. X23_MR_ was estimated in Galicia at 1.82, 1.10, and 2.22 deaths per million inhabitants in 2014, 2016, and 2018, respectively. X23_MR_ were estimated in Asturias at 1.88 and 0.97 deaths per million inhabitants in 2014 and 2017, respectively. To the best of my knowledge, this is the highest described so far in scientific literature. Galicia had a total of 167,977 hives in 2018, corresponding to 5.9% of the total in Spain, with a density of 5.7 and 6.2 hives per km^2^ and per 100 inhabitants, respectively, very close to the Spanish average. Asturias had a total of 48,990 beehives in 2018, corresponding to 2.1% of the total in Spain, with a density of 4.6 and 4.8 hives per km^2^ and per 100 inhabitants respectively, values under the Spanish average. There is not a high presence of beehives in either community.

Some reasoning on the findings can be considered. The frequency of stings, and thus of subsequent allergic reactions, is dependent on geographic, environmental, and ecological factors [53]. As mentioned in the introduction, the *Vespa velutina* is an IAS first detected in the Iberian Peninsula in 2010 and its spread throughout Spain has been very rapid. Since its arrival in Spain, the species has subsequently expanded its range and was detected in the communities of Aragón, Asturias, Balearic Islands, Cantabria, Castilla y León, Castilla-La Mancha, Cataluña, Extremadura, Galicia, País Vasco, Navarra, and La Rioja.

In Galicia, the *Vespa velutina* was first seen in 2012, and since then has inhabited and spread throughout the Galician territory (29,574 km^2^) in ever-increasing numbers. Two nests were detected in 2012, 17 nests in 2013, 769 nests in 2014, 5022 nests in 2015, and 10,642 nests in 2016 [54]. Last year, in 2019, around 25,000 nests were detected and destroyed in Galicia. The extent of the situation is reflected in the number of calls received by emergency services “112 Galicia” related to *Vespa velutina*, with 4.607, 13.054, and 25.240 calls in 2015, 2016, and 2017, respectively [55]. A total of 42,901 calls related to Asian hornet incidents in the 3-year period (2015–2017), suggesting Galicia as a spot of extremely high exposure. In Asturias (10,604 km^2^), the *Vespa velutina* was first detected in 2015, and has increased its presence with a total of 488 nests in 2017, 1564 nests in 2018, and 5351 nests in 2019 [56].

One limitation of the present work is that cases involving hornets, wasps, or bees could not be separately identified, with data provided by death certificates. Statistics extracted from hospitals and poison centers are difficult to obtain, so it is very important to facilitate the sharing of this information [38]. In this context, the different news that appeared in the media should not be ignored, since they allow us to know insect-related incidents with precise details about the fatality. Between 2014 and 2018, I recorded eight fatalities in Galicia involving wasps and/or hornets, which appeared in the media. Most of them showed enough evidence to point to the Asian hornet as the cause of death. So far this year (2020), the national press, radio, and television have reported three deaths from insect stings in Galicia and one death in Asturias, pointing to the Asian hornet as the culprit. In Galicia: (i) in September, a 69-year-old man, working in the forest, was found lying on the ground two hours after leaving his house; the first hypothesis suggests that the death occurred as a consequence of an attack by an Asian hornet nest; (ii) in May, a 73-year-old man suffered a strong allergic reaction that ended his life, when he was harvesting seeds in the garden and received the attack of *Vespa velutina* nest embedded in a close-by fencing wall; and (iii) also in May, a 54-year-old man passed away after receiving a *Vespa velutina* sting while checking on an apiary near his house. An Asian hornet nest was settled inside one of the beehives. The deceased was accompanied at the time by another beekeeper. Emergency health services rushed to the scene, but there was nothing to be done to save his life. The victim was not allergic to honeybee venom. On previous occasions, he had suffered stings from common bees/wasps without significant effects; however, this time a single sting on the face was fatal in a few minutes. In Asturias, in July, a 40-year-old man died in Asturias, due to *Vespa velutina* stings. The tragic event occurred when he was mowing grass on his property, where a nest was found.

Over 100 examples of IAS that affect human health, sometimes with devastating effects on our livelihood, have been described and documented around the world [57]. In 2018, Galician doctors demanded adrenaline kits to be distributed in health centers to combat *Vespa velutina* stings, and the Galician Health Service (SERGAS) implemented a new and specific quick route of care for people who had a generalized reaction after being sting by a Hymenoptera [58]. Presently, this invasive species generated concern among Spanish allergologists on the Cantabrian coast and in Galicia. There is currently an increase in the number and severity of reactions in patients exposed to insect venom, mainly due to the invasive alien species *Vespa velutina*. Introduction of this new species has impacted the number of hymenoptera stings seen in clinical toxicology units. Seventy-seven percent of the patients seen who had systemic allergic reactions had been stung by the Asian hornet [59]. There might be a relevant number of unidentified venom allergy-related deaths as many fatal reactions following insect stings probably remain misinterpreted and undetected [60]. The true prevalence of mortality induced by contact with hornets, wasps, and bees may be underestimated [26,27].

## 5. Conclusions

The deaths resulting from contact with hornets, wasps, and bees (ICD-10 code X23) in Spain, both at the state and sub-state level (1999–2018), were examined. Over the 20-year period studied, a total of 78 fatalities were recorded and annual mortality rates varied from 0.02 to 0.19 per 1 million inhabitants, placing Spain at low levels compared to other countries. The majority of deaths occurred in males (85.9%), in the 65-or-over age group (52.6%), at “unspecified places” (67.9%), and in the months of July and August (50%). At the sub-state level, the X23 deaths were more highly concentrated in the communities of Galicia (35.8%), Andalucía (21.7%), and Castilla y Leon (12.8%), with 28, 17, and 10 victims, respectively. A more detailed and specific breakdown of the distribution of the deaths due to stinging insects by year and across communities reveals some striking findings. Galicia showed high X23_MR_ values with 1.82 (in 2014), 1.10 (in 2016), and 2.22 (in 2018) per million inhabitants. X23_MR_ were estimated in Asturias at 1.88 and 0.97 deaths per million people in 2014 and 2017, respectively. To the best of my knowledge, these are values never found before in the available literature. One limitation of the present work is that cases involving hornets, wasps, or bees could not be separately identified, with data provided by death certificates. In this context, the different news that appeared in the media should not be ignored, since they allow to raise awareness of insect-related incidents with precise details about the fatality. Between 2014 to 2018, I recopilated eight fatalities happened in Galicia which appeared in mass media and where wasps/hornets were always involved, showing enough evidence in most cases to signal the Asian hornet as the insect causing the death. So far this year (2020), three deaths in Galicia and one in Asturias have been reported by mass media. Given the scope of the effects of the Asian hornet, it has a definitely much wider impact than just for honeybees and their keepers. Due to its habits, abundance, and broader distribution, the risk that *Vespa velutina* poses to human health is unmatched by other native species. Although administration have minimized the consequences of stings on human health at the beginning of the invasion, the presence of the invasive species *Vespa velutina*, which has settled and spread widely in the north of Spain, deserves special attention and management by the authorities. The research conducted here is useful to health administrators and policy planners in prioritizing areas with high mortality due to Hymenoptera insects. In light of these findings, there is evidence to consider the *Vespa velutina* with high medical importance and consequences, and to abandon the notion that the invasive species poses no greater risk to human health than a bee. Insect-venom anaphylaxis remains a major challenge for clinicians. More research is needed to identify the source of this variation and its relationship to different possible explanations. 

## Figures and Tables

**Table 1:** Number of deaths due to hornet, wasp, and bee stings according to place and age in Spain, 1999–2018.

Variable	Description	*n*	%
Place of occurrence	Home	6	7.6
	Residential institution	-	-
	School, other institution and public administrative area	1	1.2
	Sports and athletics	-	-
	Street and highway	4	5.1
	Trade and services	-	-
	Industry and construction	-	-
	Farm	7	8.9
	Other specified places	7	8.9
	Unspecified place	53	67.9
Age	Children (≤14 years)	-	-
	Youth (15–24 years)	-	-
	Adults (25–64 years)	37	47.4
	Seniors (≥65 years)	41	52.6

**Table 2:** Annual mortality rates due to hornet, wasp, and bee sting in Spain, at the sub-state level, 1999–2018.

Community	1999	2000	2001	2002	2003	2004	2005	2006	2007	2008	2009	2010	2011	2012	2013	2014	2015	2016	2017	2018	1999–2018
Andalucía	0.14	-	0.41	0.40	-	0.13	-	0.13	-	-	-	0.12	-	0.12	0.12	0.12	0.12	-	0.24	0.12	0.11
Aragón	-	-	-	-	0.81	-	-	-	-	-	-	-	-	-	-	-	-	-	-	-	0.04
Asturias	-	-	-	-	-	-	-	-	-	-	-	-	-	-	-	1.88	-	-	0.97	-	0.14
Balearic Islands	-	-	-	-	-	-	-	-	-	-	0.91	-	-	-	-	-	-	-	-	0.89	0.09
Canary Islands	-	-	-	-	-	-	-	-	-	-	-	-	-	-	-	-	-	-	-	-	-
Cantabria	-	-	-	-	-	-	-	-	-	-	-	-	-	-	-	-	-	-	-	-	-
Castilla y León	0.80	0.40	-	0.40	0.40	-	-	0.40	0.40	-	0.78	-	-	-	0.40	-	-	-	-	-	0.20
Castilla-La Mancha	-	-	-	-	-	-	-	-	-	-	-	0.48	0.95	-	-	-	-	-	-	0.49	0.10
Cataluña	-	-	-	-	-	0.15	-	-	-	-	-	-	-	-	-	-	-	-	-	-	0.01
Valencia	-	0.24	-	0.23	-	-	0.43	-	-	-	-	-	-	-	-	-	-	0.20	-	-	0.06
Extremadura	-	0.94	0.93	-	0.93	-	-	-	-	-	-	-	-	-	-	-	-	-	-	-	0.14
Galicia	-	-	-	-	0.73	-	0.72	-	-	0.36	0.36	-	0.72	0.72	-	1.82	0.73	1.10	0.74	2.22	0.51
Madrid	-	-	-	-	-	-	-	0.17	-	-	-	-	-	-	-	-	-	-	-	-	0.01
Murcia	-	-	-	-	-	-	-	-	-	-	0.69	-	-	-	-	-	-	-	-	-	0.03
Navarra	-	-	-	-	-	-	-	-	-	-	-	-	-	-	-	-	-	-	-	-	-
País Vasco	-	-	-	-	-	-	-	-	-	-	0.46	-	-	-	-	-	-	-	-	-	0.02
La Rioja	-	-	-	-	-	-	-	-	-	-	-	-	-	-	-	-	-	-	-	-	-
City of Ceuta	-	-	-	-	-	-	-	-	-	-	-	-	-	-	-	-	-	-	-	-	-
City of Melilla	-	-	-	-	-	-	-	-	-	-	-	-	-	-	-	-	-	-	-	-	-

**Table 3:** Mortality rates due to hornet, wasp, and bee sting reported in literature for different countries. The X23_MR_ are expressed as fatalities per year and per 1,000,000 inhabitants.

Country	Time Period	Years of Study	X23_MR_	Reference
England and Wales	1959–1971	13	0.09	Somerville et al. (1975) [44]
USA	1950–1959	10	0.14	Parrish (1963) [45]
USA	1962–1982	21	0.16	Nall (1985) [46]
USA	1991–2001	11	0.18	Langley (2005) [47]
Germany	1979–1983	5	0.18	Przybilla and Ring (1985) [48]
Denmark	1960–1980	21	0.25	Mosbech (1983) [49]
France	1981–1991	12	0.43	Charpin et al. (1994) [27]
Switzerland	1961–1963	3	0.45	Muller (1985) [50]
Costa Rica	1985–2006	22	0.74	Prado et al. (2009) [42]
Sweden	1975–1984	10	0.2	Johansson et al. (1991) [51]
Spain	1999–2018	20	0.09	Feás (2021) [present work]

## Data Availability

The data presented in this study is available on request from the Spanish National Statistical Institute.
